# The Cause of China’s Haze Pollution: City Level Evidence Based on the Extended STIRPAT Model

**DOI:** 10.3390/ijerph19084597

**Published:** 2022-04-11

**Authors:** Jingyuan Li, Jinhua Cheng, Yang Wen, Jingyu Cheng, Zhong Ma, Peiqi Hu, Shurui Jiang

**Affiliations:** 1 School of Economics and Management, China University of Geosciences, Wuhan 430074, China; ljy420@cug.edu.cn (J.L.); chengjinhua100@126.com (J.C.); 2 Chinese Academy of Macroeconomic Research, Beijing 100038, China; wenyangycywlj@163.com; 3 Institute of Spatial Planning & Regional Economy, National Development and Reform Commission, Beijing 100038, China; 4 School of Physical Education, China University of Geosciences, Wuhan 430074, China; cjy@cug.edu.cn; 5 School of Environment and Natural Resources, Renmin University of China, Beijing 100872, China; zhongma@vip.sina.com; 6 Department of Forest and Conservation Science, University of British Columbia, Vancouver, BC V6T 1Z4, Canada

**Keywords:** PM_2.5_, influencing factors, STIRPAT model, quantile regression

## Abstract

Based on the extended STIRPAT model, this paper examines social and economic factors regarding PM_2.5_ concentration intensity in 255 Chinese cities from 2007 to 2016, and includes quantile regressions to analyze the different effects of these factors among cities of various sizes. The results indicate that: (1) during 2007–2016, urban PM_2.5_ concentration exhibited declining trends in fluctuations concerning the development of the urban economy, accompanied by uncertainty under different city types; (2) population size has a significant effect on propelling PM_2.5_ concentration; (3) the effect of structure reformation on PM_2.5_ concentration is evident among cities with different populations and levels of economic development; and (4) foreign investment and scientific technology can significantly reduce PM_2.5_ emission concentration in cities. Accordingly, local governments not only endeavor to further control population size, but should implement a recycling economy, and devise a viable urban industrial structure. The city governance policies for PM_2.5_ concentration reduction require re-classification according to different population scales. Cities with large populations (i.e., over 10 million) should consider reducing their energy consumption. Medium population-sized cities (between 1 million and 10 million) should indeed implement effective population (density) control policies, while cities with small populations (less than 1 million) should focus on promoting sustainable urban development to stop environmental pollution from secondary industry sources.

## 1. Introduction

**Figure 1:**
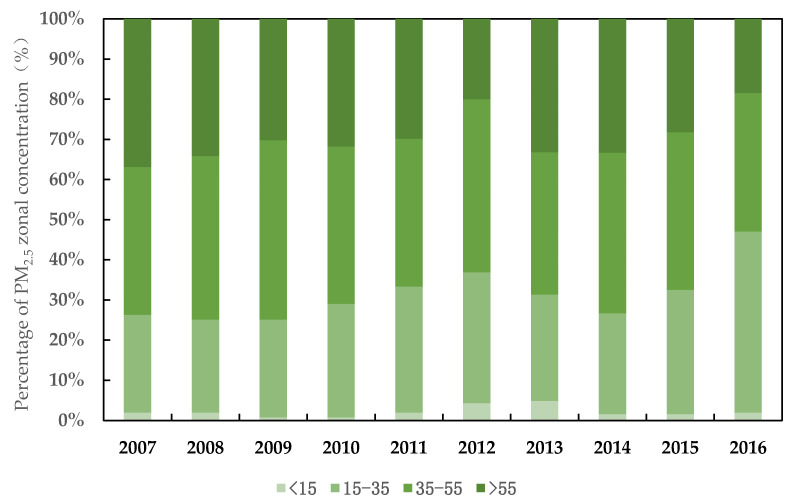
The trend regarding PM_2.5_ concentration in China’s 255 cities, 2007–2016 (μg/m^3^).

With the rapid changes occurring in industrialization and urbanization, China’s economic development has been expanding in recent decades. Consequently, massive haze pollution has been generated from such rapid economic development and it can no longer be ignored. In response to the frequent and widespread haze pollution problem, the Chinese government is now paying much greater attention to it. The Action Plan for the Prevention and Control of Air Pollution was issued in 2013, aiming to reduce the concentration of fine particles. PM_2.5_ is one of the most widespread pollutants among fine particles. The Ministry of Environmental Protection adopted the Ambient Air Quality Standards (GB3095-2012) in 2012. It stipulates the annual average concentration limit of PM_2.5_, with the primary limit standard of 15 μg/m^3^ and the secondary limit standard of 35 μg/m^3^. As seen in Figure 1, the proportion of PM_2.5_ annual average concentration below the secondary concentration limit experienced an increase from 26.27% in 2007 to 36.86% in 2012, followed by a sharp decline from 31.32% in 2013, and a steady increase up to 47.06% in 2016. The proportion of PM_2.5_ annual average concentration is higher than 55 μg/m^3^ but did decline gradually to 36.86% in 2007, then 18.43% in 2016 after a slight rebound in 2010 (31.76%) and 2013 (33.21%). The national average of PM_2.5_ concentration was 47 μg/m^3^ in 2016, with a 29% decrease compared to 2013.

Most current studies in China have stated the main pollutants of air pollution such as: carbon dioxide (CO_2_), nitrogen oxide (NO_X_), sulfur dioxide (SO_2_) and fine particulate matter (PM_2.5_) [1,2,3,4]. Dong et al. examined key impact factors, specifically the CO_2_ emissions of 128 countries from 1990 to 2014. The results indicate that causality varies among the variables across regions [5]. Diao et al. studied the industrial NOx emissions in China’s provinces, and pointed out that the government must develop appropriate regulatory policies to combat regional pollution [6]. Miao et al. focused on the key factors of dust and SO_2_ emissions and found that pollutant emissions are correlated across regions [7]. Yang and Shan also investigated regional industrial SO_2_ emissions in the Jiangsu Province and its south, middle and north regions during 2004–2016 [8]. Meanwhile Weng et al. explored the urban environmental effect driven by multi-factors, such as industrial SO_2_, wastewater and dust emission intensity in prefecture-level cities [9]. PM_2.5_ is one of the main particulate matters found in haze, which is evidenced as the main contributor of air pollution [10]. Wang et al. and Chen et al. also pointed out that PM_2.5_ can in fact seriously affect ambient air quality [11,12].

Most Chinese scholars utilize certain variables to measure population density [13], urbanization [14] and FDI [15] when discussing the driving factor of PM_2.5_ concentration at the city level. Furthermore, energy-related indicators also play an important role in PM_2.5_ concentration but the effects are rarely examined due to the lack of data captured and in cities [16], which has led to the omitted variable bias. This study uses electricity consumption to measure the level of energy consumption mainly based on the following two considerations. Firstly, electricity consumption has been widely used by scholars as an indicator since it highly correlates with urban energy consumption. Secondly, data accuracy and availability of this indicator reduces the bias of omitted variables, thus improving the reliability of results [17,18].

Scholars have used different research methods, such as exploratory spatial data analysis (ESDA) [19], factor analysis [20], the Stochastic Impacts by Regression on Population, Affluence and Technology (STIRPAT) model [21], GeogDetector [22,23] and spatial econometric model to explore the main factors affecting PM_2.5_ concentration [4]. Zhang et al. used spatial regression to mainly discuss the direct and indirect influences which several factors make on PM_2.5_ concentration. The study finds that environmental regulations not only have a direct impact on haze pollution, but also affect haze pollution indirectly through coal consumption, as it relates to foreign direct investment (FDI), and industrial structure and technological innovation [24]. Lu et al. employed both linear regression and grey system correlation analysis methods to analyze the spatial and temporal patterns, variation trends and the main factors influencing PM_2.5_ concentration in China from 1998 to 2014. The results conclude that high PM_2.5_ concentration in the northwest of China were mainly caused by natural factors (sand and dust), while in the eastern region mainly resulted from human activities. Low PM_2.5_ concentration areas were mainly located in the less developed regions [25]. Yang et al. applied the GeogDetector method to quantify nonlinear associations between PM_2.5_ concentration and potential factors. Both natural and socioeconomic factors and their interactive impacts on PM_2.5_ pollution are considered [22]. Wang et al. employed multi-temporal and statistical analysis to show the temporal and spatial characteristics of PM_2.5_ concentration in 338 Chinese cities during 2014–2017, and further used structural equation modeling (SEM) to quantify the socio-economic driving factors of PM_2.5_ concentration changes. This concluded that urban population density contributes most significantly [26]. Zhou et al. verified that population scale wields a significantly positive effect on PM_2.5_ concentration in 199 cities of China [27]. 

Few scholars evaluated the determinants of PM_2.5_ concentration among various population scales. The factors causing the problem of PM_2.5_ concentration vary in regions with different population sizes. Although previous scholars studied PM_2.5_ concentration mainly at the national level, it has gradually expanded to provide research into the regions and regional or cross-regional levels, such as the Yangtze River Delta region, Beijing-Tianjin-Hebei region and various provinces of China [28,29,30]. For instance, Luo et al. employed an extended STIRPAT model to identify the socioeconomic determinants of PM_2.5_ concentration for 12 different regions in China, and ranked the influencing factors on PM_2.5_ concentration in descending order of importance [31]. Cheng et al. used the epsilon-based measure (EBM) meta-frontier Malmquist model to measure the meta-frontier Malmquist total factor productivity index (MMPI) in 10 city groups [32]. According to the above studies, most research focused on the differentiated determinants of PM_2.5_ concentration in various geographic regions.

To differentiate the diverse effects of population sizes on PM_2.5_ concentration, the quantile regression method is implemented. The ordinary least squares (OLS) method only analyzes the expected value of the effect of each explanatory variable. It cannot ascertain the influence of each factor on the distribution patterns alone of PM_2.5_ concentration and distinguish the different results from one factor using such varied types. Quantile regression as proposed by Koenker and Bassett (1978), however, can perhaps solve this problem [33]. The quantile regression method assumes the quantile of the conditional distribution of the dependent variable as a linear function, and thus establishes a quantile regression of the dependent variable. It can obtain the quantile effect of the independent variable on the dependent variable, which gives a more detailed picture of the conditions of distribution and a more comprehensive set of results. It can avoid the shortcomings of OLS regression in analyzing the outliers and heteroskedasticity of the dependent variable [34,35]. Consequently, it is essential to consider population scale in our research by using the quantile regression model.

The Environmental Kuznets Curve (EKC) theory is another important issue widely discussed in current literatures. The inverted U-shaped curve relationship between the level of environmental pollution and income level implies that environmental degradation increases with output during the early stages of economic growth. However, it declines with output after reaching a specified threshold. Some scholars further studied the EKC hypothesis and found that haze pollution and economic development not only have an inverted U-shaped curve relationship [36,37], but also have a U-shaped [38], N-shaped or inverted N-shaped relationship [39,40]. While some regions could not confirm the EKC hypothesis [41,42]. Apergis et al. postulated that the EKC hypothesis holds in only 10 states, while the remaining 38 states do not find the EKC hypothesis, which should prevent environmental degradation but not at the expense of economic growth [43]. Current studies such as those above have analyzed the driving factor of PM_2.5_ concentration using various models to support the existence of EKC in PM_2.5_ concentration. As referred to previously, a few scholars have combined the influential elements on PM_2.5_ concentration and the EKC of the economy and PM_2.5_ concentration, which is worth investigating further. 

In summary, three aspects contribute to this research field. Firstly, instead of only considering the geological differences, cities in China are categorized into four types to explore the differentiated effect of variables on PM2.5 concentration classified by population scale. Secondly, the urban scale and EKC effect are both taken into account. This paper constructs the STIRPAT model of PM_2.5_ concentration in 255 cities and combines the EKC hypothesis to measure the determinants of haze pollution more comprehensively. Thirdly, total electricity consumption as a critical proxy of energy consumption is also involved in the model to measure the influence of energy consumption on PM_2.5_ concentration, which enriches the model with more variables.

The following is further discussed. Section 2 presents the methodology, variables and data sources. Section 3 represents the results of the STIRPAT model and quantile regression. Section 4 proposes further discussion. The corresponding conclusions are articulated in Section 5.

## 2. Materials and Methods

### 2.1. Methodology

In 1971, Ehrlich and Holden first proposed the “*I* = *PAT*” model [44] to study the relationship between population and environment. Dietz later proposed the Stochastic Impacts by Regression on Population, Affluence and Technology (STIRPAT) model which takes the general form:

(1) $${I=\alpha P}^{b}A^{c}T^{d}$$

where I refers to environmental impact, P denotes population size, A represents affluence, T denotes technology level and *e* denotes random disturbance term. Both sides of the equation are taken logarithmically as:

(2) ln(I)=α+bln(P)+clnA+dlnT+e

To measure the influence of energy on urban PM_2.5_ concentration, an extended STIRPAT-based model is implemented to empirically analyze the influencing factors of PM_2.5_ concentration in 255 cities in China. The EKC curve between PM_2.5_ emission intensity and economic development is also investigated. The equation in the form of logarithm is as follows:

(3) $$\begin{array}{c} \ln\left({\mathrm{PM}}_{2.5\mathrm{it}}\right){=\alpha }_{0}{+\alpha }_{1}\ln\left({\mathrm{PD}}_{\mathrm{it}}\right){+\alpha }_{2}\ln\left({\mathrm{GDP}}_{\mathrm{it}}\right){+\alpha }_{3}\ln{\left({\mathrm{GDP}}_{\mathrm{it}}\right)}^{2}\\ {+\alpha }_{4}\ln{\left({\mathrm{GDP}}_{\mathrm{it}}\right)}^{3}{+\alpha }_{5}\ln\left({\mathrm{SE}}_{\mathrm{it}}\right){+\alpha }_{6}\ln\left({\mathrm{TEC}}_{\mathrm{it}}\right){+\alpha }_{7}\ln\left({\mathrm{FI}}_{\mathrm{it}}\right){+\alpha }_{8}\ln\left({\mathrm{PSP}}_{\mathrm{it}}\right){+\mu }_{\mathrm{it}} \end{array}$$

where *i* represents region, *t* stands for time, *PM*_2.5_ is PM_2.5_ concentration in region *i* at time *t*, *PD* denotes population density, *GDP* denotes gross domestic product, *SE* refers to science expenditure, *TEC* denotes total electricity consumption, *FI* indicates actual foreign investment amount, *PSP* denotes the proportion of secondary industry in GDP and *μ_it_* denotes the disturbance term.

In order to investigate the influence of PM_2.5_ concentration in cities with different population sizes at different quartiles, the following quantile regression model is developed.

(4) $${\mathrm{Quant}}_{\theta }\left((\ln\left({\mathrm{PM}}_{2.5\mathrm{it}}\right){)|X}_{\mathrm{it}}\right){=\beta }^{\theta }X_{\mathrm{it}}\;$$

In Equation (4), *X_it_* is the independent variable; *β^θ^* is the coefficient vector; and ${\mathrm{Quant}}_{\theta }\left((\ln\left({\mathrm{PM}}_{2.5\mathrm{it}}\right){)|X}_{\mathrm{it}}\right)$ denotes the conditional quantile of PM_2.5_ concentration corresponding to the quantile *θ* (0 < *θ* < 1) for a given X. The coefficient vector *β^θ^* corresponding to θ is achieved by minimizing the absolute deviation (LAD):

(5) $$\begin{array}{c} \beta^{\theta }=\mathrm{argmin}\left\{\underset{i,\;t,\;(\ln\left({\mathrm{PM}}_{2.5\mathrm{it}}\right))\geq X_{\mathrm{it}}\beta }{\sum }\theta \left|(\ln\left({\mathrm{PM}}_{2.5\mathrm{it}}\right){)-X}_{\mathrm{it}}\beta \right|\right\}\;\\ +\underset{i,\;t,\;(\ln\left({\mathrm{PM}}_{2.5\mathrm{it}}\right){)<X}_{\mathrm{it}}\beta }{\sum }\left(1-\theta \right)\left|(\ln\left({\mathrm{PM}}_{2.5\mathrm{it}}\right){)-X}_{\mathrm{it}}\theta \right|\}\; \end{array}$$

The bootstraps intensive algorithm technique is applied to estimate the quantile regression coefficients $\beta^{\theta }$, which means that the confidence interval of the sample is obtained by continuously having a put-back sampling process.

### 2.2. Variable and Data

Integrating domestic and foreign studies, PM_2.5_ concentration is adopted as the proxy variable of air pollution, which can directly represent the extent of haze pollution. Six explanatory variables are selected in this paper based on the STIRPAT model. The main explanatory variables in STIRPAT model include: (1) Population density (*PD*) is the number of people in per unit area. (2) Gross domestic product (*GDP*) measures the level of urban output. This paper uses 2007 as the base period to do the treatment of the invariant price. (3) Science expenditure (*SE*) represents the intensity of government R&D investment. The control variables are selected according to the existing literature using extended STIRPAT model. They include: (1) total electricity consumption (*TEC*) used to measure the degree of energy consumption [45]; (2) actual foreign investment (*FI*) calculated to measure the degree of openness to the world [46]; and (3) the share of secondary industry in GDP (*PSP*) used to measure the effect of industrial structure on PM_2.5_ concentration [47].

In addition, two effects are considered in this paper due to the complexity of PM_2.5_ emission concentration impacts:

(1) Urban scale effect. Population is a crucial indicator of city characteristics, which not only reflects the level of urban development, but also is one of the main factors affecting PM_2.5_ concentration. Therefore, this paper adopts the variable of population as the classification criterion and divides 255 cities into four categories of cities, i.e., four groups of dummy variables, according to the Notice of the State Council on Adjusting the Criteria of City Size Classification issued by the State Council in 2014. Of these, cities with a population size of more than 10 million are Type I cities (Type I cities are the reference group), cities with populations between 5 million and 10 million are Type II cities, cities with populations between 1 million and 5 million are Type III cities, and cities with populations less than 1 million are Type IV cities. In the following analysis, the influence of each four city categories on PM_2.5_ concentration will be reported. The definitions and descriptive statistics of all the above variables are shown in Table 1.

(2) EKC effect. The EKC effect is taken into consideration to verify the relationship between haze pollution and GDP. Before applying it, most studies based on a particular sample of data do not examine the random characteristics of the data and determine their suitability for the model. This brings about the deviation in the curve from the sample points within the sample interval [47]. We also introduce the secondary and tertiary terms of GDP into the model. The shape of the EKC curve is judged according to the coefficients $\alpha_{2}$, $\alpha_{3}$ and $\alpha_{4}$ of Equation (3).

In order to explore the factors influencing PM_2.5_ concentration in cities in China, 255 sample cities were selected for the period 2007–2016. To ensure that the logarithm value is positive, all ratios are calculated as percentages. The presence of large differences among variables makes the use of logarithms rational. Data sources are as follows. The PM_2.5_ concentration data of each prefecture-level city were obtained from the global raster data based on satellite monitoring published by the Center for Socioeconomic Data and Applications of Columbia University. Other data were obtained from China Urban Statistical Yearbook (2008–2017), China Environmental Statistical Yearbook (2008–2017) and the provincial statistical yearbooks in (2008–2017).

## 3. Results

### 3.1. Stationarity Test

To avoid the problem of pseudo-regressions and invalid *t*-tests, the Levin–Lin–Chu (LLC) test [48] and Fisher-ADF test [49] assess the stationarity of the variables (see Table 2). Except *lnPD* for horizontal series, all the results in Table 2 passed the significance test with a *p*-value less of than 0.01. Moreover, all variables for first-difference series are stationary at the 1% significance level.

According to the co-integration theory, if all variables are single integers of the same order, co-integration relationships may exist between variables. Based on the Eangle and Granger two-step method, the Kao co-integration test of homogeneity and Padroni co-integration test of heterogeneity are used [50,51,52]. Table 3 shows the results of different statistics in the Kao test and Pedroni test, which all passed the significance test with *p*-value less than 0.01. Hence, long-term stable equilibrium relationships exist among PM_2.5_ concentration, foreign direct investment, electricity consumption, GDP, population density, share of secondary industry and science expenditure. A mixed OLS regression is illustrated in Table 2.

### 3.2. Extended STIRPAT Model of 255 Cities

Based on the panel data of 255 cities in China from 2007–2016, a mixed OLS regression is conducted. Both the random effect and the fixed effect are considered in panel data analysis. A Hausman test is adopted for modeling selection (See in Table 4). The result of the Hausman test shows significance, which shows evidence for the fixed effects model being more applicable than the random effects model. In addition, the F-test results of the fixed effects model demonstrate that the fixed effects model is better than the OLS regression, strongly suggesting that it is more appropriate to use fixed effects in the econometric regression.

Column 3 in Table 4 shows the fixed effect of ordinary least squares regression. For the main explanatory variables in the expanded STIRPAT model, *lnPD* (coefficient 0.172) passes the significance test at the 1% level, which means that for every 1% rise in the population density, PM_2.5_ concentration increases by 0.172%. The coefficients of *lnGDP* and (*lnGDP*)^3^ are 0.558 and 0.003, respectively, statistically significant at the 5% and 10% levels. Furthermore, the coefficient of (*lnGDP*)^2^ is −0.08 at the 10% significance level. It indicates an inverted N-shaped relationship between PM_2.5_ concentration and GDP, confirming the existence of the Kuznets curve. Based on the trends of PM_2.5_ concentration in 255 cities from 2007 to 2016, the inflection points of PM_2.5_ concentration in each city will occur when economic progress is made. The coefficient of *lnSE* is −0.048 at the 1% significance level, illustrating that the PM_2.5_ concentration will increase by 0.048% when the scientific expenditure decreases by 1%. As for the control variables, the coefficients of *lnTEC* (0.011) and *lnFI* (0.011) are, respectively, statistically significant at the 10%, and 1% levels, which means that both total electricity consumption and foreign direct investment function to promote PM_2.5_ concentration in 255 Chinese cities. The PM_2.5_ concentration rises by 0.011% when total electricity consumption and actual use of foreign direct investment expands by 1%. On the contrary, *lnPSP* (coefficient −0.058) is negative at the 5% level, which shows that the share of secondary production will lead to a decline in PM_2.5_ concentration.

### 3.3. Quantile Regression by Different Population Size

The estimated coefficients in the expanded STIRPAT model can reflect the overall situation for all cities. Considering substantial city samples and long observation times in this paper, the overall OLS regression is not able to reflect the heterogeneity of the influential factors in various city categories based on population scale. A quantile regression is carried out to distinguish the differential effects of influential indicators on the PM_2.5_ concentration for cities by various population-size categories and different quantile of city-size categories in every city size. The quantile regression model is an essential reference for the formulation and piloting implement of PM_2.5_ concentration control policies in different cities. It is regressed that based on the conditional quantile of the dependent variable, the conditional quantile describes the variation of each indicator on PM_2.5_ concentration more accurately. The 25%, 50% and 75% quantile represent the different PM_2.5_ concentration from low to high in the same type cities. For example, the 25%, 50% and 75% quantile in type I mean the lower, middle and higher PM_2.5_ concentration cities in this type, respectively.

**Figure 2:**
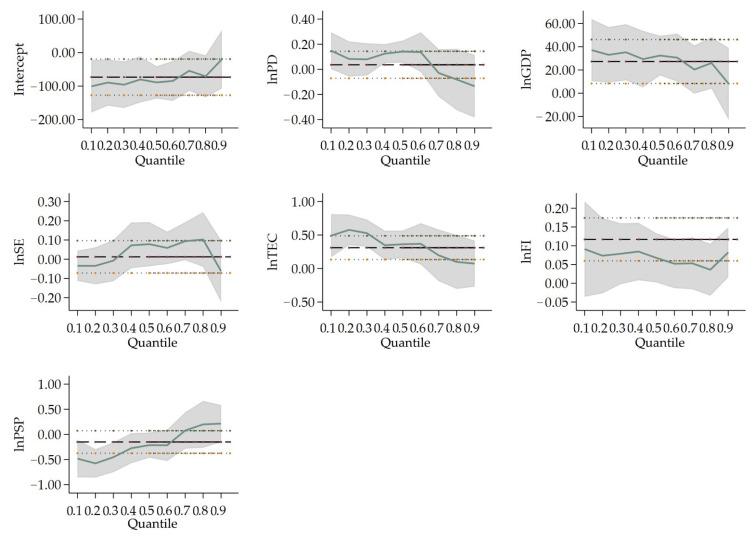
Change in variables’ coefficients in Type I cities’ quantile regression. Notes: The thicker dashed lines indicate the OLS regression estimates for the independent variables; the area which is parallel with thinner dashed lines is the confidence interval (95% confidence level) for the regression coefficients; The solid lines are the quantile regression coefficients of the respective variables, and the shaded area is the confidence interval (95% confidence level) of the quantile regression analysis estimates. The horizontal axis is the quantile of the dependent variable; the vertical axis is the regression estimate of the independent variable.

**Figure 3:**
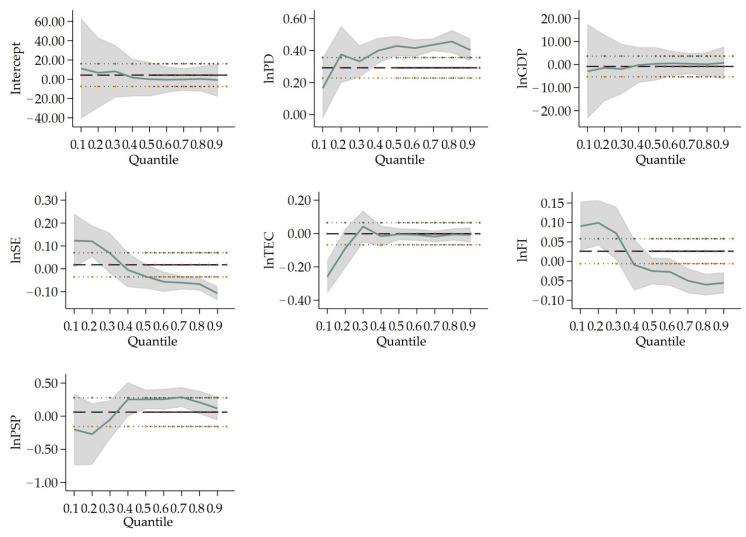
Change in variables’ coefficients in Type II cities’ quantile regression. Notes: the labels for all lines, the horizontal and vertical axis are the same as above.

**Figure 4:**
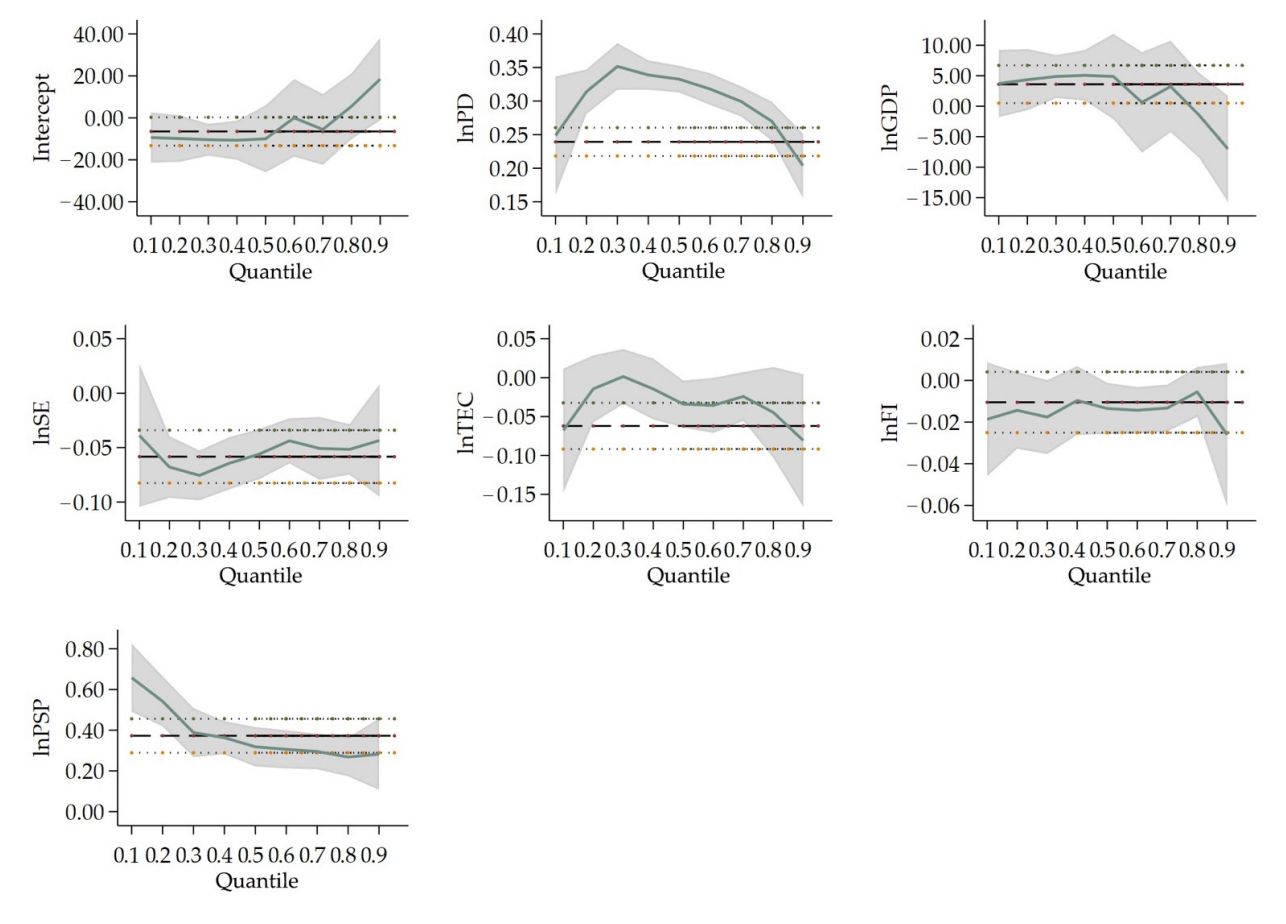
Change in variables’ coefficients in Type III cities’ quantile regression. Notes: the labels of all lines, the horizontal and vertical axis are the same as above.

**Figure 5:**
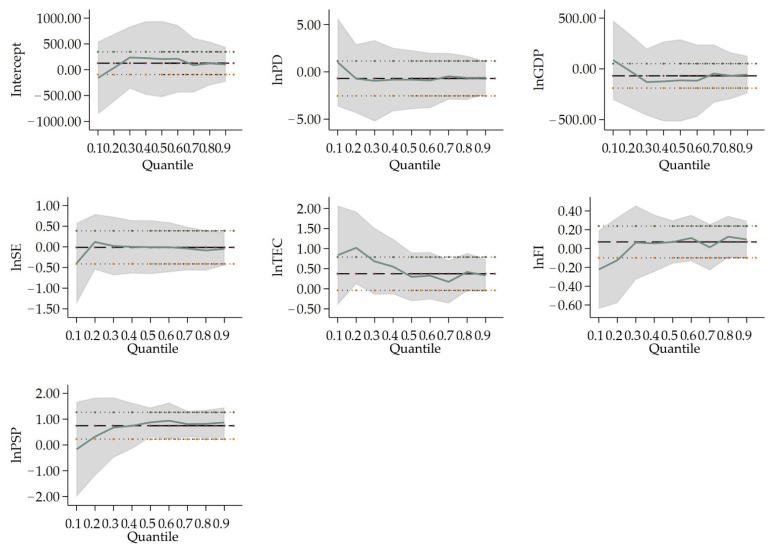
Change in variables’ coefficients in Type IV cities’ quantile regression. Notes: the labels for all lines, the horizontal and vertical axis are the same as above.

Table 5 shows the results of the quantile regression in four city-size categories. Figure 2, Figure 3, Figure 4 and Figure 5 show the changes in the variables’ coefficients in four types of cities’ quantile regressions. In Type I cities (population larger than 10 million), the coefficient of *lnPD* (0.141) is only significant at 10% significance when tested by the 50% quantile regression. This illustrates that population density worsens air pollution only for the middle PM_2.5_ concentration cities. *lnGDP*, (*lnGDP*)^2^ and (*lnGDP*)^3^ are significant at 25%, or 50% quantile regression, the coefficients of which are positive, negative and positive at the 1% significance level. It indicates an inverted N-type Kuznets curve in cities with low and medium PM_2.5_ concentration. This is consistent with the national situation. The coefficients of *lnTEC* are 0.557, 0.363 and 0.166, respectively, at 25%, 50% and 75% quantile regression. However, only coefficients at 25%, or 50% quantile pass the 1% significance level. This indicates that in Type I cities, the effect of energy consumption on air pollution is weakening as the PM_2.5_ concentration rises. The coefficients of *lnFI* and *lnPSP* at 25% quantile regression are 0.087 and −0.549, which pass the significance test at 10% and 1%, respectively. It indicates that for every 1% increase in foreign direct investment, haze pollution is raised by 0.087%. Otherwise, PM_2.5_ concentration rather declines by 0.549% as the share of secondary production advances every 1%. 

In Type II cities (population size between 5 million and 10 million), *lnPD* passes the significance test at 1% level, the coefficients of which are 0.353, 0.428 and 0.451 at 25%, 50% and 75% quantile regression, respectively. Such results illustrate that the enhanced population density does accelerate PM_2.5_ concentration in Type II cities. For instance, the higher the PM_2.5_ concentration is, the stronger the effect of population density on haze pollution is. The coefficient of *lnSE* is significantly negative at 25% and 75% quantile, which is 0.091 and −0.057, respectively. *lnFI* are significant at 25%, 50% and 75% quantile regression. The coefficient at 25% quantile is positive (0.096), while the coefficients at 50% and 75% quantile are both negative (−0.025 and −0.056, respectively). It indicates that in Type II cities, the science expenditure and foreign direct investment will accelerate PM_2.5_ concentration at lower PM_2.5_ concentration cities, but hinder the PM_2.5_ concentration at higher PM_2.5_ concentration cities. *lnPSP* are significant at 1% and 5% level, respectively, the coefficients of which are −0.254 at 50% quantile and −0.234 at 75% quantile. These results indicate that the share of secondary production will exert a negative influence on air pollution at middle and higher PM_2.5_ concentration cities in Type II. 

In Type III cities (population between 1 million and 5 million), the coefficients of *lnPD* all pass the significance test at the 1% level, which are 0.343, 0.333 and 0.289 at 25%, 50% and 75% quantile regression, respectively. These results illustrate that population density will increase PM_2.5_ concentration in Type III cities. However, the higher the PM_2.5_ concentration is, the weaker the effect of population density on haze pollution. *lnGDP*, (*lnGDP*)^2^ and (*lnGDP*)^3^ are significant at 25% quantile regression, and the coefficients of which are 3.766, −0.524 and 0.025, in fact indicate the existence of a N-type Kuznets curve in cities with lower PM_2.5_ concentration. The coefficients of *lnSE* are −0.068, −0.056 and −0.052, which all pass the significance test at the 1% level. Expenditure on science will encourage technological innovation, which helps to improve the end-treatment measures and reduce polluting emissions and can to some extent improve air quality. Only *lnTEC* at 50% quantile regression passes the significance test in Type III. Total electricity consumption can alleviate air pollution only at middle PM_2.5_ concentration cities. The coefficients of *lnFI* are −0.019 and −0.013 at 25%, 50% quantile regression, respectively, which pass the 5% significance level. PM_2.5_ concentration will abate by 0.019% when foreign direct investment falls by 1% in cities with lower PM_2.5_ concentration. For middle PM_2.5_ concentration cities, PM_2.5_ concentration drops by 0.013% when foreign direct investment decreases by 1%. This indicates that in Type III cities, increases in foreign direct investment will restrict air pollution. The coefficients of *lnPSP* are 0.421, 0.319 and 0.279, all passing the significance test at the 1% level. The share of secondary production does exacerbate haze pollution, and the influence will grow as the PM_2.5_ concentration becomes higher in Type III cities.

In Type IV cities (population size less than 1 million), only the coefficient of *lnPSP* (0.876) at 50% quantile is significant, which means that the secondary production will exacerbate air pollution in cities with small population size. 

## 4. Discussion

### 4.1. The Discussion of Expanded STIRPAT Model Results

**Figure 6:**
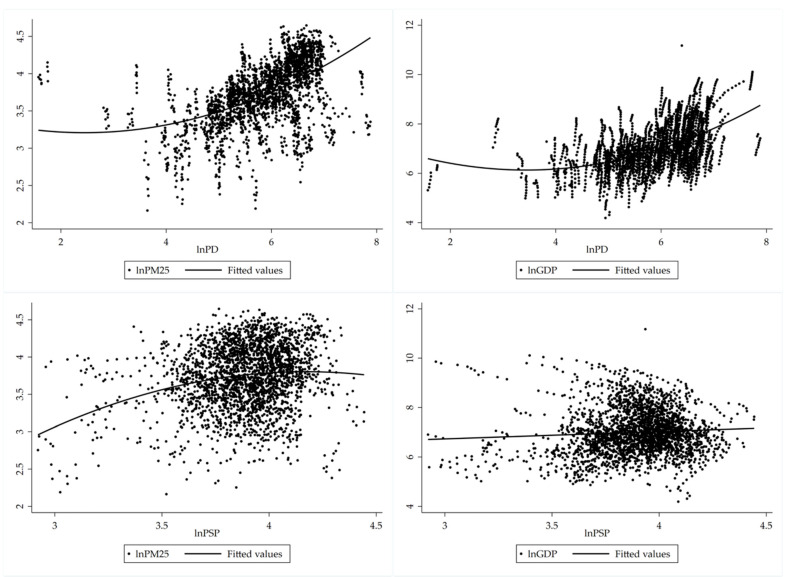
The relationship between PM2.5 concentration and population density and secondary industry.

On a national scale, population density exerts the greatest positive influence on PM_2.5_ concentration. During urbanization, having a much larger and concentrated population is one of the important manifestations. The relationship between PM_2.5_ concentration and population density is shown in Figure 6. Along with the increase in urban population density, the *GDP* of cities also rises, which brings about the increment in PM_2.5_ concentration. Cities with different urban population scales have significant diverse effects on PM_2.5_ concentration. PM_2.5_ concentration in Type I (cities over 10 million people), Type II (cities between 5 million and 10 million individuals) and Type III cities (cities between 1 million and 5 million people) are significantly higher compared to Type IV (cities less than 1 million people) cities, as can be seen in Table 3. 

Social economic activities in cities with larger populations are more frequent, thus the PM_2.5_ concentration are correspondently higher. The economic growth has an inverted N-shaped EKC curve with air pollution, which indicates the following experiences in Chinses cities. First, urban economic progress is accompanied by a reduction in haze pollution. Then, the higher economic expansion jeopardizes the environment and increases emissions. Finally, the air pollution gradually declines again when further economic development is accomplished. Expenditure on science has a significant effect on PM_2.5_ concentration reduction. Research into pollution reduction technology and on haze emission reductions to improve industries can effectively mitigate urban PM_2.5_ concentration. 

Meanwhile, the coefficient of *lnTEC* (0.011) means that electricity consumption surges by every 1% when PM_2.5_ concentration rises by 0.011%, due to the high correlation between society-wide electricity consumption and energy consumption. Foreign direct investment exerts a significant positive effect on PM_2.5_ concentration, i.e., the PM_2.5_ concentration rises by 0.011% when the actual utilization of foreign direct investment expands by 1%. This shows that foreign direct investment will accelerate haze pollution emissions, indicating that FDI inflow contributed to air pollution and the “pollution shelter” hypothesis was established in China. From the perspective of industrial distribution in foreign direct investment in 2015, the secondary industry accounts for the most of it, mainly the manufacturing (31.32%) and real estate (22.96%) industries. The unabated expansion of these industries and their current polluting processes will certainly compromise the quality of urban air. 

On the contrary, the ratio of secondary industry to the regional GDP has a significant negative effect on PM_2.5_ concentration for the following reasons. At the outset, the proportion of secondary industry is closely related to the level of urban economic development. When cities’ secondary industry expands, urban economic development increases. Secondly, as the latter increases (see Figure 2), cities with a comparatively high level of economic sophistication will gradually eliminate backward production methods and move high-polluted and energy-consuming industries to less developed vicinities. Greener and more intensive and sustainable industries will take their place. Thus, the pollution generated by these cities will be transferred to underdeveloped areas. 

### 4.2. The Differences between Driving Indicators on Cities according to Different Population Sizes

When it comes to the effect of two main variables (population density (*lnPD*) and GDP (*lnGDP*, (*lnGDP*)^2^, (*lnGDP*)^3^)) on different population sizes, the population density of Type I cities only influences PM_2.5_ concentration at the 50% quantile, indicating that in megacities with more than 10 million people, the population density does not show a significant effect on PM_2.5_ concentration. On the other hand, this indeed enhances PM_2.5_ concentration in Type II and III cities, for the reason that the regression coefficients of population density are all significantly positive at 25%, 50% and 75% quantile in these two types. In the middle-sized cities, the influx of residents accelerates urbanization and the consumption of private cars and houses, resulting in the rise of PM_2.5_ concentration. The coefficients of economic development are significant at the 25% and 50% quantile in Type I cities. In Type I cities, there is an inverted N-type Kuznets curve in cities with lower and medium PM_2.5_ concentration cities. In general, population density plays a more important role on PM_2.5_ concentration in Type II and III cities, while economic development has a more crucial effect on PM_2.5_ concentration in Type I cities comparatively.

Considering the perspective of industrial structure and energy consumption, in Type I cities, PM_2.5_ concentration is influenced more by energy consumption than other types of cities. It is shown that the coefficients of total electricity consumption are significant at the 25% and 50% quantile regression in Type I cities. Meanwhile, the coefficients of total electricity consumption are not significant in Type II and illustrate being negative for Type III. PM_2.5_ concentration enlarges as energy consumption increases in low and medium emission cities in Type I cities. Meanwhile, the proportion of the secondary industry can make a positive impact on PM_2.5_ concentration for Type II and III cities. Secondary industry expands accompanied by a surge in resource consumption of cities with permanent residents between 1 million to 10 million, which brings about the increment of PM_2.5_ concentration. In Type IV cities, the coefficient of industrial structure in the 75% quantile is the only significant variable. Cities with comparatively higher emissions in this type are influenced more by the secondary industry. Pollution will continue once the related manufacturing develops for those cities with a small scale of population.

Furthermore, foreign direct investment and science expenditure have different effects on PM_2.5_ concentration both in different types of cities and at different quantile regressions. In Type I cities, the foreign direct investment only boosts the PM_2.5_ concentration forming the 25% quantile at 10% significance level. As for Type II cities, the coefficients of foreign direct investment are 0.096, −0.025 and −0.056 at 25%, 50% and 75% quartile, while those of science expenditure are 0.091, −0.033 and−0.057 at 25%, 50% and 75% quartile. As the PM_2.5_ concentration increases in Type II cities, the effect of both foreign direct investment and science expenditure on PM_2.5_ concentration are moving from positive to negative with less populated cities to higher PM_2.5_ concentration cities. Type II cities with more than 5 million permanent residents tend to gradually transfer pollutants from higher PM_2.5_ concentration cities to lower PM_2.5_ concentration cities. This indicates that pollution is transferred to the lower PM_2.5_ concentration cities along with the transfer of foreign investment in most Chinese cities. Concerning Type III cities, both foreign direct investment and science expenditure have negative effects on PM_2.5_ concentration, but their effectiveness is much slighter compared to the strong rise in PM_2.5_ concentration from population density and the secondary industry. Foreign direct investment and science expenditure are required for Type III cities which aim to improve production equipment and processes, and abate pollutant emissions. 

## 5. Conclusions

This paper analyzes the driving factors of PM_2.5_ concentration in China’s 255 prefecture-level cities from 2007 to 2016 based on the expanded STIRPAT model. The differences of each influencing factor across city categories are compared, while the differences of various influencing factors within each city category are compared using the quantile regression.

The main conclusions of this paper are as follows. On the whole, a inverted N-shaped Kuznets curve between economic development and PM_2.5_ concentration exists in China. The phenomena of “pollution shelter” have been revealed in 255 of China’s cities, and the progress of technology will improve the air quality. Meanwhile, the effects of driving factors on air pollution vary between cities with different population scales. Population density plays a more dominant role in PM_2.5_ concentration in Type II and III cities (medium population between 1 million and 10 million), and energy consumption has a more crucial effect on PM_2.5_ concentration in Type I cities (large populations over 10 million) comparatively. The secondary industry will enhance the PM_2.5_ concentration in most Type IV cities (small population less than 1 million).

The above research conclusion provides policy recommendations for air pollution abatement in cities as follows. Firstly, policymakers should treat the relationship between environmental emission reduction and economic development rationally. From a national aspect, an EKC relationship exists between GDP and the environment; however, from the perspective of cities of different sizes, the EKC curve does not really exist between GDP and the environment. The relationship between economic development and air pollution in megacities is in line with the law of the Kuznets curve, and at the peak of the haze pollution. The medium and large population-sized cities coexist with the scale effect and technology effect, and the peak of haze pollution is not yet ascertained. The pollution in medium and large population-sized cities is influenced by changes in population size, so medium cities are in fact considered as diseconomies of scale caused by the expansion of population size when economic progress occurs. Consequently, policymakers should help balance the population size of cities with economic development, and pay more attention to the environment, technological improvement and better use of local natural resources. 

Secondly, the proportion of the secondary industry has a great influence on urban PM_2.5_ concentration, it is necessary to reasonably determine the scale of urban development and city size, promote industrial transformation and upgrade small and medium-sized cities synergistically in order to promote their functioning, state of industry and the environment, and promote the green economy and circular economy. Energy consumption has a more significant impact on the air quality of megacities; thus, policymakers should improve the concept of green consumption by enterprises and residents.

Thirdly, government should emphasize science and technology instead of focusing on better targeted investment. This is especially the case when introducing green capital to improve the situation of “pollution shelter” and increase investment in research on how to reduce pollution and improve remediation strategies.

## Figures and Tables

**Table 1:** Data sources and definition of variables and descriptive statistics.

Variable	Definition	Units of Measurement	Mean	Median	Standard Deviation	Minimum	Maximum
*PM*_2.5_	PM_2.5_ emissions concentration	$µg/m^{3}$	45.71	43.02	18.09	8.70	104.30
*PD*	Population density	$\mathrm{Per}\;\mathrm{person}/{\mathrm{km}}^{2}$	459.15	393.21	332.59	4.82	2648.11
*GDP*	Per capita gross domestic product	10,000 Yuan	1786.00	991.03	2722.38	66.13	71,340.28
*SE*	Scientific expenditures	10,000 Yuan	70,652.13	18,797.00	238,982.50	469	4,035,240.00
*TEC*	Total electricity consumption	Billion kWh	156.41	102.59	175.52	2.25	1486.02
*FI*	Foreign investment	10,000 Dollars	84,956.71	22,596	196,498.10	16.00	3,082,563.00
*PSP*	Ratio of secondary industry to GDP	%	49.86	50.16	9.67	18.57	85.08

**Table 2:** Unit root tests of variables.

Unit Root Tests	Variable	LLC	Fish-ADF
Horizontal Sequence	*lnPM*_2.5_	−15.4620 ***	13.4164 ***
	*lnPD*	−3.3360 ***	−1.2033
	*lnGDP*	−49.3459 ***	47.0489 ***
	*lnSE*	−18.9008 ***	6.3799 ***
	*lnTEC*	−20.5336 ***	5.1772 ***
	*lnFI*	−12.8356 ***	5.6464 ***
	*lnPSP*	−3.7413 ***	2.9823 ***
First difference	*lnPM*_2.5_	−2.0958 ***	5.2782 ***
	*lnPD*	−15.2374 ***	9.6203 ***
	*lnGDP*	−38.8361 ***	16.4137 ***
	*lnSE*	−31.7062 ***	13.7043 ***
	*lnTEC*	−82.8749 ***	59.3129 ***
	*lnFI*	−60.2082 ***	30.7214 ***
	*lnPSP*	4.9806 ***	9.6083 ***

Notes: ***, ** and * represent significance at the 1%, 5% and 10% levels, respectively.

**Table 3:** Co-integration tests of variables.

Test Method	Statistics	Statistics Value
Kao test	ADF	−18.9077 ***
Pedroni test	Panel PP	−57.0751 ***
	Panel ADF	−42.8427 ***

Notes: ***, ** and * represent significance at the 1%, 5% and 10% levels, respectively.

**Table 4:** Regression results of factors influencing urban PM_2.5_ concentration.

Variable	OLS	Fixed Effects	Random Effects
(1)	(2)	(3)	(4)
*lnPD*	0.220 ***	0.172 ***	0.164 ***
	(0.025)	(0.048)	(0.029)
*lnGDP*	0.523 **	0.558 **	0.563 **
	(0.264)	(0.226)	(0.262)
(*lnGDP*)^2^	−0.075 **	−0.080 **	−0.081 **
	(0.036)	(0.036)	(0.036)
(*lnGDP*)^3^	0.003 *	0.003 *	0.003 **
	(0.0016)	(0.002)	(0.002)
*lnSE*	−0.051 ***	−0.048 ***	−0.047 ***
	(0.006)	(0.004)	(0.006)
*lnTEC*	0.013	0.011 *	0.011
	(0.009)	(0.006)	(0.009)
*lnFI*	0.012 ***	0.011 ***	0.011 ***
	(0.003)	(0.001)	(0.003)
*lnPSP*	−0.059 **	−0.058 **	−0.060 **
	(0.026)	(0.026)	(0.026)
*Isize*2		0.487 ***	0.491 ***
		(0.013)	(0.185)
*Isize*3		0.668 ***	0.678 ***
		(0.045)	(0.190)
*Isize*4		0.872 ***	0.886 ***
		(0.064)	(0.213)
*cons*	1.973 ***	1.622 **	1.667 **
	(0.656)	(0.570)	(0.670)
*Hausman test*	46.07 *** (Prob > chi = 0.000)		
The shape of EKC	N-shaped		

Notes: ***, ** and * represent significance at the 1%, 5% and 10% levels, respectively.

**Table 5:** Quantile regression results of factors influencing urban PM_2.5_ concentration.

Variable	Type I	Type II	Type III	Type IV
(1)	(2)	(3)	(4)	(5)
*QR*_25				
*lnPD*	0.064	0.353 ***	0.343 ***	−1.184
	(0.059)	(0.072)	(0.022)	(2.066)
*lnGDP*	36.893 ***	−0.508	3.766 **	−132.832
	(12.299)	(5.852)	(1.806)	(187.802)
(*lnGDP*) ^2^	−4.319 ***	−0.031	−0.524 *	24.446
	(1.412)	(0.772)	(0.279)	(34.582)
(*lnGDP*) ^3^	0.164 ***	0.004	0.025 *	−1.499
	(0.054)	(0.034)	(0.014)	(2.124)
*lnSE*	−0.035	0.091 ***	−0.068 ***	0.050
	(0.058)	(0.031)	(0.014)	(0.459)
*lnTEC*	0.557 ***	−0.032	−0.007	0.808
	(0.112)	(0.054)	(0.018)	(0.482)
*lnFI*	0.087 *	0.096 ***	−0.019 **	0.088
	(0.050)	(0.024)	(0.008)	(0.190)
*lnPSP*	−0.549 ***	−0.203	0.421 ***	0.637
	(0.186)	(0.160)	(0.090)	(0.599)
_*cons*	−100.563 ***	4.447	−8.302 **	243.430
	(35.434)	(14.793)	(3.936)	(342.007)
*QR*_50				
*lnPD*	0.141 *	0.428 ***	0.333 ***	−0.824
	(0.078)	(0.032)	(0.013)	(1.343)
*lnGDP*	32.408 ***	0.333	4.885	−113.113
	(11.645)	(3.207)	(3.304)	(108.114)
(*lnGDP*) ^2^	−3.818 ***	−0.036	−0.705	20.916
	(1.355)	(0.427)	(0.483)	(19.736)
(*lnGDP*) ^3^	0.146 ***	0.001	0.034	−1.287
	(0.052)	(0.019)	(0.023)	(1.201)
*lnSE*	0.078	−0.033	−0.056 ***	−0.007
	(0.065)	(0.028)	(0.011)	(0.295)
*lnTEC*	0.363 ***	−0.005	−0.034 ***	0.295
	(0.110)	(0.023)	(0.012)	(0.289)
*lnFI*	0.068	−0.025 *	−0.013 **	0.070
	(0.043)	(0.014)	(0.006)	(0.152)
*lnPSP*	−0.214	0.254 ***	0.319 ***	0.876 **
	(0.153)	(0.088)	(0.057)	(0.334)
*_cons*	−88.946 ***	0.104	−9.985	206.373
	(33.277)	(7.979)	(7.455)	(198.116)
*QR*_75				
*lnPD*	−0.052	0.451 ***	0.289 ***	−0.390
	(0.130)	(0.027)	(0.021)	(0.910)
*lnGDP*	20.410	−0.174	−0.567	−67.150
	(12.892)	(3.215)	(3.391)	(81.754)
(*lnGDP*) ^2^	−2.412	0.058	0.050	12.531
	(1.515)	(0.432)	(0.498)	(14.908)
(*lnGDP*) ^3^	0.092	−0.004	−0.000	−0.778
	(1.515)	(0.432)	(0.498)	(14.908)
*lnSE*	0.096	−0.057 ***	−0.052 ***	−0.093
	(0.080)	(0.017)	(0.014)	(0.209)
*lnTEC*	0.166	−0.014	−0.032	0.309
	(0.188)	(0.016)	(0.021)	(0.231)
*lnFI*	0.054	−0.056 ***	−0.007	0.104
	(0.043)	(0.014)	(0.009)	(0.126)
*lnPSP*	0.111	0.234 **	0.279 ***	0.939 ***
	(0.197)	(0.092)	(0.057)	(0.219)
_*cons*	−54.477	1.314	3.475	120.487
	(36.651)	(7.956)	(7.660)	(149.651)

Notes: ***, ** and * represent significance at the 1%, 5% and 10% levels, respectively. ^2^ and ^3^ represent the quadratic and cubic power of *lnGDP* respectively.

## Data Availability

The basic data used in the research can be found on the website of the National Bureau of Statistics, China Statistical Yearbook and other databases.
